# Factors associated with blood pressure control in patients with hypertension and HIV at a large urban HIV clinic in Uganda

**DOI:** 10.1038/s41371-022-00786-7

**Published:** 2022-12-07

**Authors:** Douglas Joseph Musimbaggo, Isaac Derick Kimera, Christabellah Namugenyi, Jeremy I. Schwartz, Rebecca Ssenyonjo, Fortunate Ambangira, Lubega Kizza, Mary Mbuliro, Rodgers Katwesigye, Isaac Ssinabulya, Martin Muddu, Dinesh Neupane, Michael Hecht Olsen, Manan Pareek, Fred C. Semitala

**Affiliations:** 1 Makerere University Joint AIDS Program, Kampala, Uganda; 2 Department of Medicine, Yale New Haven Hospital, Yale School of Medicine, New Haven, CT USA; 3 Department of Medicine, Makerere University College of Health Science, Kampala, Uganda; 4 Uganda Heart Institute, Kampala, Uganda; 5 Johns Hopkins Bloomberg School of Public Health, Baltimore, MD USA; 6 Department of Regional Health Research, University of Southern Denmark, Odense, Denmark; 7 Cardiology Section, Department of Internal Medicine, Holbaek Hospital, Holbaek, Denmark

**Keywords:** Hypertension, Risk factors

## Abstract

Globally, people living with HIV on antiretroviral therapy have an increased risk of cardiovascular disease. Hypertension is the most important preventable risk factor for cardiovascular disease and is associated with increased morbidity. We conducted an exploratory survey with hypertensive persons living with HIV who received integrated HIV and hypertension care in a large clinic in Uganda between August 2019 and March 2020 to determine factors associated with blood pressure control at six months. Controlled blood pressure was defined as <140/90 mmHg. Multivariable logistic regression was used to determine baseline factors associated with blood pressure control after 6 months of antihypertensive treatment. Of the 1061 participants, 644 (62.6%) were female. The mean age (SD) was 51.1 (9.4) years. Most participants were overweight (*n* = 411, 38.7%) or obese (*n* = 276, 25.9%), and 98 (8.9%) had diabetes mellitus. Blood pressure control improved from 14.4% at baseline to 66.1% at 6 months. Comorbid diabetes mellitus (odds ratio (OR) = 0.41, 95% confidence interval (CI) = 0.26–0.64, *p* < 0.001) and HIV status disclosure (OR = 0.73, 95% CI = 0.55–0.98, *p* = 0.037) were associated with the absence of controlled blood pressure at 6 months. In conclusion, comorbid diabetes mellitus and the disclosure of an individual’s HIV status to a close person were associated with poor blood pressure control among persons living with HIV who had hypertension. Therefore, subpopulations of persons living with HIV with hypertension and comorbid diabetes mellitus may require more thorough assessments and intensive antihypertensive management approaches to achieve blood pressure targets.

## Introduction

Globally, persons living with human immunodeficiency virus (HIV) who are on antiretroviral therapy (ART) have a high risk of cardiovascular disease (CVD) [1]. Compared with HIV-negative individuals, persons living with HIV are twice as likely to present with a cardiovascular event [2]. Sub-Saharan Africa (SSA) accounts for over 80% of the global burden of persons living with HIV [3]. Increased coverage of ART in SSA has improved patient management and contributed to reduced AIDS-related mortality [4]. However, improved survival has also led to an increase in comorbidities due to noninfectious causes such as hypertension [5]. Furthermore, hypertensive persons living with HIV are at increased risk of CVD morbidity and mortality compared with those without hypertension [6]. Both HIV and hypertension are associated with chronic inflammation, immune activation and endothelial dysfunction, which further contribute to the increased CVD risk. HIV itself, however, contributes to a state of persistent inflammation, immune activation, vascular dysfunction and lipodystrophy. These affect a number of pathways, including microbial translocation, dyslipidemias, renin-angiotensin-aldosterone systems and HIV-associated renal disease, that later contribute to the development of hypertension [7]. Persons living with HIV and hypertension have unique challenges, such as increased pill burden, drug‒drug interactions and frequent clinical reviews, and these unique challenges may affect early blood pressure control. The prevalence of hypertension in adult persons living with HIV in Uganda is estimated at 20-25% [8, 9], which is comparable to that of 26.5% among the general population [10].

To address the growing burden of hypertension among persons with HIV in SSA, the World Health Organization (WHO) and individual national HIV guidelines have recommended the integration of hypertension and HIV care [11, 12]. Despite such efforts in SSA, most integration programs have reported hypertension control rates below that of 50% recommended by the WHO [13]. Achievement of hypertension control within six months is a predictor of reduced cardiovascular morbidity and mortality [14], but there is a paucity of data on rates of and factors associated with blood pressure control in HIV patients receiving integrated care.

In this study, we aimed to determine factors associated with blood pressure control at 6 months among hypertensive persons living with HIV receiving integrated hypertension and HIV care in a large urban HIV clinic in Uganda.

## Methods

### Study design and setting

This was an exploratory survey among persons living with HIV who were treated at the Mulago ISS Clinic from August 2019 to March 2021. The Mulago ISS Clinic is the largest HIV clinic in Uganda and provides comprehensive HIV services to more than 16,500 active patients. The clinic is located within the Mulago National Referral and Teaching Hospital Complex in Kampala, Uganda. HIV services include HIV counseling and testing and ART management. The clinic also implements differentiated ART delivery models; in these models, stable patients with good adherence to ART and sustained HIV viral suppression receive their ART refills in the community through community client-led ART delivery and community drug distribution points and at the facility through fast track drug refills. The Mulago ISS clinic is owned and operated by the Makerere University Joint AIDS Program (MJAP). The HIV care services are supported with funding from President’s Emergency Plan For AIDS Relief. At this clinic, services are provided by doctors, nurses, clinical officers, HIV counselors, laboratory technicians, pharmacy technicians, records officers and expert patients (people living with HIV who have openly declared their status) [15]. The services are provided at no cost to the patients.

### Study population

We included persons living with HIV who were ≥18 years old, were enrolled in HIV care at the clinic and were receiving integrated HIV and hypertension care. In line with the national guidelines for HIV care (Consolidated Guidelines for the Prevention and Treatment of HIV and AIDS in Uganda), patients are routinely screened for noncommunicable diseases, including hypertension [12]. With funding from Resolve to Save Lives, the Mulago ISS clinic integrated the management of hypertension into HIV care beginning in August 2019. All patients were screened for hypertension as recommended by the HIV guidelines, and those diagnosed with hypertension provided verbal consent before enrollment in the integrated HIV-hypertension care program. We collected data between August 2019 and March 2020 (the first 6 months of integrated care). This study was approved by the institutional review board of The AIDS Support Organization (TASO) and The Uganda Council for Science and Technology (UNCST). Persons living with HIV were enrolled when they were diagnosed with hypertension and consented to receive care under the integrated care program. When an individual is diagnosed with hypertension, a clinician typically prescribes both ART and antihypertensive medications at the same time and schedules the patient for the review of both conditions at a future appointment. Prior to the commencement of integrated care, the Mulago ISS clinic had already achieved universal screening for hypertension among people living with HIV at each clinic visit.

### Data collection

The clinic uses a combination of paper files and an electronic medical record (EMR) system to record data on integrated hypertension and HIV care documented at each clinic visit by the clinician who evaluates the patient. We reviewed the two data sources to extract data that were collected during routine clinic visits of hypertensive people living with HIV for the following data elements: sociodemographic characteristics, clinical measurements, laboratory findings, and prescribed antihypertensive medications, including ART.

### Main exposures, outcomes, and definitions

The objective of this analysis was to explore factors associated with blood pressure control after 6 months of integrated hypertension and HIV care. Controlled blood pressure was defined as an office blood pressure <140/90 mmHg as per the World Health Organization (WHO), the European Society of Cardiology (ESC)/European Society of Hypertension (ESH) and the International Society of Hypertension (ISH) adopted for the Uganda Clinical Guidelines. Blood pressure was measured after five minutes of rest while the patient was in a sitting position with both the back and hand supported. For patients with a blood pressure>140/90 mmHg, two additional measurements were taken with a two-minute interval, and the lowest reading was documented using Omro M6 (HEM-7321-E) validated automated blood pressure machines at the baseline and follow-up visits [16]. Hypertensive people living with HIV received monthly medicine refills for both HIV and hypertension, with stepwise titration of hypertensive medicines until their blood pressure was controlled. Amlodipine, valsartan and hydrochlorothiazide were the three medicines used. Patients were given amlodipine as the first-line antihypertensive medicine, followed by valsartan and hydrochlorothiazide arranged on a treatment protocol to guide dosage titration. The maximum doses of the medicines were as follows: 10 mg of amlodipine, 160 mg of valsartan and 25 mg of hydrochlorothiazide. The 6-month observation period was selected based on evidence that early blood pressure control (within 6 months) is beneficial for the prevention of major cardiovascular events. Chronic kidney disease was defined as an estimated glomerular filtration rate (eGFR) <60 ml/min/1.73 m^2^ calculated using the Modification of Diet in Renal Disease (MDRD) equation [17].

Diabetes mellitus was defined as a single fasting plasma glucose level ≥7 mmol/L or the use of anti-diabetic medication [18].

Body mass index (BMI) was calculated from height and weight using the following standard formula: BMI = weight (kg)/height^2^ (m^2^). Standard cut offs were used for categorization: <18.5 kg/m^2^ was defined as underweight, 18.5-24.9 kg/m^2^ was normal weight, 25–29.9 kg/m^2^ was overweight, and ≥ 30 kg/m^2^ was considered obese.

### Statistical analysis

We analyzed data for persons living with HIV who had completed six months of integrated hypertension and HIV care beginning in August 2019. Participant characteristics were stratified for uncontrolled versus controlled blood pressure and are presented as counts and percentages. Groupwise differences were assessed using Pearson’s chi-squared test (when all cell counts were ≥5) or Fisher’s exact test (when at least one cell count was <5). All variables were included in a multivariable binary logistic regression analysis because they were thought to be important in predicting blood pressure control at 6 months. Odds ratios (ORs) with 95% confidence intervals (CIs) were reported. We also conducted a multivariable linear regression analysis to determine the association between the different covariates and absolute blood pressure changes from baseline to 6 months. A two-sided *P* value < 0.05 was considered significant. No adjustment for multiplicity was made, as the analyses were considered exploratory. We used Stata version 14 (StataCorp LP, College Station, TX, USA) for all analyses.

## Results

Data from 1061 persons living with HIV were available at both baseline and 6 months. At baseline (Table 1), most participants were female (*n* = 664, 62.6%) with a mean age of 51.1 (SD 9.4) years, and only 153 participants (14.4%) had controlled blood pressure. The mean systolic blood pressure was 158 mmHg (SD 18.7), and the mean diastolic blood pressure was 101 mmHg (SD 12.0). A family history of hypertension was reported for 796 patients (74.9%). Ninety-six (8.9%) of the participants had diabetes mellitus. The majority of the study subjects were overweight (*n* = 411, 38.7%) or obese (*n* = 276, 25.9%), and the mean BMI was 27 (SD 5.2) kg/m^2^. One hundred forty-three (13.5%) patients with controlled blood pressure at baseline had received ART for more than five years. Ninety-one (8.6%) patients were active smokers, and 43 (4.1%) patients had a history of stroke. Only 13 (1.2%) patients had chronic kidney disease. Almost all participants (*n* = 1050, 99.1%) had a suppressed viral load (<1000 copies/ml).

**Table 1:** Baseline characteristics of the participants who received integrated HIV and hypertension care at the Mulago ISS clinic.

Variable	Uncontrolled hypertension at baseline (*n* = 908)	Controlled hypertension at baseline (*n* = 153)	Total (*N* = 1061)	*p* value
Sex: *n* (%)				0.822
Female	567 (62.4)	97 (63.4)	664 (62.6)	
Systolic BP (mean, SD)	161.3 (17.3)	139.2 (15.4)	158.1 (18.7)	<0.001
Diastolic BP (mean, SD)	102.4 (11.4)	89.7 (9.1)	100.6 (12.0)	<0.001
Age group (years): n (%)				0.110
<30	4 (0.3)	0 (0.0)	4 (0.4)	
30–39	95 (10.5)	10 (6.5)	105 (9.8)	
40–49	324 (35.0)	45 (29.4)	369 (34.8)	
≥50	485 (54.2)	98 (64.1)	583 (55.1)	
Active smoking: *n* (%)	81 (9.0)	10 (6.5)	91 (8.6)	0.310
Family history of hypertension: *n* (%)	674 (74.3)	122 (79.7)	796 (74.9)	0.153
Diabetes mellitus: *n* (%)	74 (8.3)	22 (14.4)	96 (8.9)	0.150
Prior myocardial infarction: *n* (%)	2 (0.2)	0 (0.0)	2 (0.2)	0.681
Prior stroke: *n* (%)	39 (4.0)	4 (2.6)	43 (4.1)	0.417
Chronic kidney disease: *n* (%)	12 (1.3)	1 (0.7)	13 (1.2)	0.487
BMI (mean, SD)	27.8 (5.7)	26.8 (5.1)	27.0 (5.2)	0.020
BMI (kg/m^2^) categories: *n* (%)				0.442
Underweight (<18.5)	30 (3.6)	2 (1.3)	32 (3.1)	
Normal weight (18.5–24.9)	291 (31.9)	51 (33.3)	342 (32.3)	
Overweight (25–29.9)	354 (39.0)	57 (37.3)	411 (38.7)	
Obese (≥30)	233 (25.4)	43 (28.1)	276 (25.9)	
HIV status disclosure: *n* (%)	451 (49.7)	76 (49.7)	527 (49.5)	0.899
Baseline ART regimen: *n* (%)				0.340
TDF/3TC/DTG	25 (2.6)	1 (0.7)	26 (2.5)	
AZT/3TC/NVP	380 (41.9)	64 (41.8)	444 (41.8)	
AZT/3TC/EFV	154 (16.9)	35 (22.9)	189 (17.8)	
TDF/3TC/NVP	81 (9.3)	16 (10.5)	97 (9.2)	
TDF/3TC/EFV	226 (24.9)	31 (20.3)	257 (24.1)	
ABC/3TC/DTG	19 (2.2)	2 (1.3)	21 (2.0)	
Others	23 (2.3)	4 (2.6)	27 (2.6)	
ART duration (years): *n* (%)				0.074
<5	87 (9.0)	10 (6.5)	97 (9.2)	
5–10	395 (43.4)	81 (52.3)	476 (44.8)	
≥10	426 (47.6)	62 (41.2)	488 (46.1)	
Baseline CD4 (cells/µl): *n* (%)				0.633
<50	149 (16.4)	20 (13.1)	169 (15.9)	
50–99	60 (6.5)	13 (8.5)	73 (6.8)	
100–199	220 (24.5)	38 (24.8)	258 (24.5)	
≥200	479 (52.6)	82 (53.6)	561 (52.78)	
Suppressed viral load (<1000 copies/ml): *n* (%)	898 (99.0)	152 (99.4)	1050 (99.1)	0.689

*ART* antiretroviral therapy, *ABC* abacavir, *AZT* zidovudine, *BP* blood pressure, *BMI* body mass index, *CD4* cluster of differentiation 4, *DTG* dolutegravir; *EFV* efavirenz, *HIV* human immunodeficiency virus, *NVP* nevirapine, *SD* standard deviation, *TDF* tenofovir, *3TC* lamivudine.

After 6 months (Table 2), nearly two-thirds of the participants (*n* = 701, 66.1%) had controlled blood pressure, with a mean systolic blood pressure of 130 mmHg (SD 25.1) and diastolic blood pressure of 84 mmHg (SD 16.6). Nearly half of the patients with diabetes mellitus (*n* = 46/96, 47.9%) had controlled blood pressure at 6 months, and a third of the patients who had disclosed their HIV status to a person who was close to them (*n* = 196/527, 37.2%) had controlled blood pressure at 6 months of follow-up.

**Table 2:** Characteristics of the participants who received integrated HIV and hypertension care at the Mulago ISS clinic at 6 months.

Variable	Uncontrolled hypertension at 6 months (*n* = 360)	Controlled hypertension at 6 months (*n* = 701)	Total (*N* = 1061)	*p* value
Sex: *n* (%)				
Female	226 (62.8)	438 (62.5)	664 (62.6)	0.924
Systolic BP (mean, SD)	148.3 (15.2)	120.8 (23.9)	129.6 (25.1)	<0.001
Diastolic BP (mean, SD)	96.5 (10.9)	78.3 (15.6)	84.1 (16.6)	<0.001
Age group (years): *n* (%)				0.325
<30	1 (0.3)	3 (0.4)	4 (0.4)	
30–39	41 (11.4)	64 (9.1)	105 (9.8)	
40–49	133 (36.9)	236 (33.8)	369 (34.8)	
≥50	185 (51.4)	398 (56.8)	583 (55.1)	
Active smoking: *n* (%)	35 (9.7)	56 (8.0)	91 (8.6)	0.341
Family history of hypertension: *n* (%)	281 (78.1)	515 (73.5)	796 (74.9)	0.098
Diabetes mellitus: *n* (%)	50 (13.9)	46 (6.6)	96 (8.9)	<0.001
Prior myocardial infarction: *n* (%)	0 (0.00)	2 (0.3)	2 (0.2)	0.334
Prior stroke: *n* (%)	16 (4.4)	27 (3.9)	43 (4.1)	0.655
Chronic kidney disease: *n* (%)	2 (0.6)	11 (1.6)	13 (1.2)	0.157
BMI (mean, SD)	27.1 (4.9)	26.9 (5.3)	27.0 (5.2)	0.446
BMI (kg/m^2^) categories: *n* (%)				0.248
Underweight (<18.5)	7 (1.9)	25 (3.6)	32 (3.1)	
Normal weight (18.5–24.9)	108 (30.0)	234 (33.4)	342 (32.3)	
Overweight (25-29.9)	148 (41.1)	263 (37.5)	411 (38.7)	
Obese (≥30)	97 (26.9)	179 (25.5)	276 (25.9)	
HIV status disclosure: *n* (%)	196 (54.4)	331 (47.2)	527 (49.5)	0.024
Baseline ART regimen: *n* (%)				0.373
TDF/3TC/DTG	10 (2.8)	16 (2.3)	26 (2.5)	
AZT/3TC/NVP	148 (41.1)	296 (42.2)	444 (41.8)	
AZT/3TC/EFV	63 (17.5)	126 (17.9)	189 (17.8)	
TDF/3TC/NVP	27 (7.5)	70 (9.9)	97 (9.2)	
TDF/3TC/EFV	96 (26.7)	161 (22.9)	257 (24.1)	
ABC/3TC/DTG	10 (2.8)	11 (1.7)	21 (2.0)	
Others	6 (1.7)	21 (2.9)	27 (2.6)	
ART duration (years):				0.723
<5	32 (8.9)	65 (9.3)	97 (9.2)	
5-Sep	170 (47.2)	306 (43.6)	476 (44.8)	
≥10	158 (43.9)	330 (47.1)	488 (46.1)	
Baseline CD4 (cells/µl):				0.781
<50	57 (15.8)	112 (15.9)	169 (15.9)	
50–99	26 (7.2)	47 (6.7)	73 (6.9)	
100–199	81 (22.5)	177 (25.2)	258 (24.4)	
≥200	196 (54.4)	365 (52.1)	561 (52.9)	
Suppressed viral load (<1000 copies/ml): *n* (%)	354 (98.3)	696 (99.4)	1050 (99.0)	0.103

*ART* antiretroviral therapy, *ABC* abacavir, *AZT* zidovudine, *BP* blood pressure, BMI body mass index, *CD4* cluster of differentiation 4, *DTG* dolutegravir; *EFV* efavirenz, *HIV* human immunodeficiency virus, *NVP* nevirapine, *SD* standard deviation, *TDF* tenofovir, *3TC* lamivudine.

In multivariable logistic regression analysis (Table 3), diabetes mellitus (OR = 0.41, 95% CI = 0.26–0.64, *p* < 0.001) and having disclosed HIV status (OR = 0.73, 95% CI = 0.55–0.98, *p* = 0.037) were significantly associated with poor blood pressure control at 6 months. In multivariable linear regression analysis, only a high baseline systolic blood pressure (beta coefficient 0.68, 95% CI = 0.56-0.81, *p* < 0.001) and a family history of hypertension (beta coefficient −5.17, 95% CI = −9.49 to −0.86, *p* = 0.019) were associated with blood pressure changes at 6 months (Table 4).

**Table 3:** Multivariable logistic regression model for systolic blood pressure at six months of follow-up in participants who received integrated HIV and hypertension care at the Mulago ISS clinic.

Variable	Adjusted OR [CI]	*p* value
Baseline systolic BP	0.98 [0.97, 0.99]	<0.001
Baseline diastolic BP	0.97 [0.95, 0.98]	<0.001
Sex		
Male	1.06 [0.77, 1.45]	0.709
Age group (years):		
<30	Reference	
30–39	0.64 [0.06, 7.16]	0.718
40–49	0.74 [0.07, 8.07]	0.803
** ≥**50	0.79 [0.07, 8.71]	0.851
Active smoking	0.85 [0.53, 1.37]	0.507
Family history of hypertension	0.79 [0.57, 1.09]	0.154
Diabetes mellitus	0.41 [0.26, 0.64]	<0.001
Prior stroke	0.83 [0.42, 1.64]	0.591
Chronic kidney disease	2.99 [0.56, 15.85]	0.197
BMI (kg/m^2^) categories:		
Normal weight (18.5–24.9)	Reference	
Underweight (<18.5)	1.56 [0.63, 3.85]	0.332
Overweight (25–29.9)	0.79 [0.57, 1.11]	0.178
Obese (≥30)	0.76 [0.53, 1.11]	0.161
HIV status disclosure	0.73 [0.55, 0.98]	0.037
Baseline ART regimen:		
TDF/3TC/DTG	Reference	
AZT/3TC/NVP	0.99 [0.40, 2.46]	0.996
AZT/3TC/EFV	1.41 [0.53, 3.72]	0.486
TDF/3TC/NVP	0.98 [0.40, 2.38]	0.962
TDF/3TC/EFV	0.59 [0.17, 2.05]	0.404
ABC/3TC/DTG	1.12 [0.47, 2.69]	0.798
Others	1.79 [0.49, 6.43]	0.373
ART duration (years):		
<5	Reference	
5-Sep	1.64 [0.12, 21.66]	0.705
≥10	1.25 [0.09, 16.05]	0.861
Baseline CD4 (cells/µl):		
<50	Reference	
50–99	0.79 [0.43, 1.48]	0.469
100–199	0.97 [0.62, 1.51]	0.897
≥200	0.86 [0.58, 1.27]	0.45
Suppressed viral load (<1000 copies/ml)	2.59 [0.69, 9.66]	0.156

*ART* antiretroviral therapy, *ABC* abacavir, *AZT* zidovudine, *BP* blood pressure, *BMI* body mass index, *CD4* cluster of differentiation 4, *CI* confidence interval, *DTG* dolutegravir, *EFV* efavirenz, *HIV* human immunodeficiency virus, *NVP* nevirapine, *OR* odds ratio, *TDF* tenofovir, *3TC* lamivudine.

**Table 4:** Multiple linear regression model for systolic blood pressure changes from baseline to six months of follow-up in participants who received integrated HIV and hypertension care at the Mulago ISS clinic.

Variable	Coefficient [CI]	*p* value
Baseline systolic BP	0.68 [0.56, 0.81]	<0.001
Baseline diastolic BP	0.01 [−0.17, 0.19]	0.904
Sex:		
Male	−2.32 [−6.63, 2.00]	0.293
Age group (years):		
<30	Reference	
30–39	−7.60 [−39.28, 24.07]	0.638
40–49	−9.99 [−41.32, 21.34]	0.532
** ≥**50	−11.09 [−42.42, 20.23]	0.487
Active smoking	1.16 [−5.54, 7.86]	0.734
Family history of hypertension	−5.17 [−9.49, −0.86]	0.019
Diabetes mellitus	−4.97 [−11.52, 1.59]	0.137
Prior stroke	−1.75 [−11.27, 7.76]	0.718
Chronic kidney disease	−2.31 [−19.41, 14.79]	0.791
BMI (kg/m^2^) categories:		
Normal weight (18.5–24.9)	Reference	
Underweight (<18.5)	1.94 [−9.11, 13.00]	0.730
Overweight (25–29.9)	−0.48 [−4.99, 4.04]	0.836
Obese (≥30)	−1.99 [−7.12, 3.15]	0.448
HIV status disclosure	−0.66 [−4.62, 3.30]	0.745
Baseline ART regimen:		
TDF/3TC/DTG	Reference	
AZT/3TC/NVP	−2.06 [−14.83, 10.71]	0.752
AZT/3TC/EFV	3.53 [−9.94, 17.01]	0.607
TDF/3TC/NVP	−0.97 [−13.51, 11.58]	0.880
TDF/3TC/EFV	−0.56 [−18.30, 17.18]	0.950
ABC/3TC/DTG	1.56 [−10.79, 13.92]	0.804
Others	5.01 [−11.67, 21.69]	0.556
ART duration (years):		
<5	Reference	
5–10	10.23 [−26.63, 47.08]	0.586
≥10	7.75 [−28.72, 44.22]	0.677
Baseline CD4 (cells/µl):		
<50	Reference	
50–99	−2.20 [−10.75, 6.34]	0.613
100–199	−1.08 [−7.19, 5.04]	0.729
≥200	−2.53 [−7.91, 2.84]	0.356
Suppressed viral load (<1000 copies/ml)	−2.23 [−21.24, 16.78]	0.818

*ART* antiretroviral therapy, *ABC* abacavir, *AZT* zidovudine, *BP* blood pressure, *BMI* body mass index, *CD4* cluster of differentiation 4, *CI* confidence interval, *DTG* dolutegravir, *EFV* efavirenz, *HIV* human immunodeficiency virus, *NVP* nevirapine, *SBP* systolic blood pressure, *SBP change* SBP at baseline-SBP at 6 months, *TDF* tenofovir, *3TC* lamivudine.

## Discussion

In this study, we aimed to determine factors associated with blood pressure control at 6 months among hypertensive persons living with HIV receiving integrated hypertension and HIV care in a large urban HIV clinic in Uganda. We observed significant improvement in blood pressure control from baseline to 6 months. However, persons living with HIV and diabetes mellitus and those who had disclosed their HIV status to a person who was close to them were less likely to have controlled blood pressure at 6 months. In a separate analysis, patients with diabetes mellitus and those who had disclosed their HIV status had higher baseline blood pressures and therefore did not achieve blood pressure control despite average blood pressure reductions. We also noted that patients with a family history of hypertension who did not have blood pressure control at baseline were less likely to achieve blood pressure control at six months.

Despite the generally high rate of blood pressure control, participants with diabetes mellitus were less likely to have blood pressure control at six months. Similarly, Suh et al. [19] and Mutua et al. [20] showed that the majority of patients with concomitant hypertension and diabetes mellitus did not achieve their blood pressure targets. These conditions commonly coexist, and both carry an increased risk of CVD [21]. In addition, the chronic nature of HIV infection with the advent of ART carries a significant risk for cardio-metabolic pathology [22]. This results in a complex disease pattern that could negatively affect health outcomes [23]. For example, a reduced response to antihypertensive medications is common in patients with diabetes mellitus. Diabetes mellitus also increases the risk for chronic kidney disease, and the latter is, by itself, a risk factor for resistant hypertension [24]. Furthermore, persons living with HIV and concomitant diabetes mellitus and hypertension have an increased pill burden, which could contribute to poor adherence [25]. Indeed, simpler ART regimens are associated with improved adherence and treatment outcomes. Therefore, such individuals may require targeted approaches to support them in attaining their blood pressure goals.

Surprisingly, participants who reported disclosing their HIV status to a close person were less likely to have controlled blood pressure at 6 months [26]. In a study by Atuyambe et al., most participants who disclosed their HIV status reported positive support from family members, such as encouragement of ART adherence, although a few patients who had negative experiences required ongoing psychosocial support [27]. In addition, Sanz et al. reported that psychosocial factors could account for uncontrolled blood pressure through poor treatment compliance and self-discipline. Based on the results of our study, poor blood pressure control with HIV status disclosure may be an indirect marker of ongoing psychosocial uneasiness that may require further examination.

We observed that the majority of the participants were either overweight or obese. The reasons for the high prevalence of overweight and obese people living with HIV can be attributed to the long-term effects of HIV infection and the subsequent exposure to antiretroviral therapy and low adherence to healthy lifestyles [28]. Notably, the majority of our study participants had been on ART for more than five years. Bakal et al. noted that 18% of people living with HIV are likely to develop obesity within five years of initiating ART. Furthermore, he recommended lifestyle modification, including weight control, for people living with HIV [29].

Additionally, we noted a very high rate of blood pressure control among people living with HIV and hypertension, demonstrating early success of integrated HIV and hypertension care. The integration of hypertension and HIV care has been shown to be associated with improved blood pressure control [30]. The high rate of blood pressure control in this program can be attributed to a number of factors: first, the majority of the participants had received ART for five years or more, and a long duration on ART is associated with improved medication adherence [31]. Second, routine ART adherence counseling and offering support to patients would inadvertently contribute to adherence to additional antihypertensive medications and thus improve blood pressure control [30]. Third, integrated care models, in which a patient is managed by the same clinician who addresses both conditions in one visit, are patient-centered and reduce redundant clinic visits [28]. Finally, in this clinic, patients were offered both hypertension and ART medications at no cost. Improved access to blood pressure medicines is a key pillar for hypertension control programs in primary health care, as highlighted in the WHO HEARTS technical package [32].

In an earlier study from Malawi integrating hypertension and HIV care, blood pressure control at 6 months was 38% [13]. It is thus clear that a higher rate of blood pressure control among persons living with HIV and hypertension can be attained with integrated care compared with the ∼5 to 8% rate of control in the general population. Ameh et al. reported that people living with HIV receiving integrated care were more likely to have controlled HIV and hypertension [33]. There has been an exponential increase in the burden of noncommunicable diseases in SSA over the past two decades due to increasing incidences of cardiovascular risk factors such as unhealthy diets, hypertension, reduced physical activity, obesity dyslipidemias, diabetes, and air pollution. Noncommunicable diseases are set to overtake communicable, maternal, neonatal, and nutritional diseases combined as the leading cause of mortality in SSA by 2030 [34]. With the already existing success of the WHO public health approach to HIV treatment and care that emphasizes task sharing, decentralization, and the integration of HIV services with other public health programs, and also with the growing knowledge base of the successful integration of HIV and hypertension care, African health ministries and their implementing partners ought to leverage the existing HIV public health programs to integrate management for noncommunicable diseases.

Our study had some limitations. Data on HIV status disclosure were self-reported without objective validation. In addition, our analysis focused on patients who had received integrated hypertension and HIV care from the clinic without considering participants who received care from alternative facilities whose characteristics may have differed from our study population. Third, we did not consider out-of-office blood pressure, which is a good predictor of sustained blood pressure control; however, we acknowledge the importance of white coat hypertension and the need for more research considering out-of-office blood pressure in our settings. However, we included all patients who had received integrated HIV and hypertension care at the clinic, and our findings can thus be generalized to this population.

Data related to chronic noncommunicable diseases along the continuum of HIV care are scarce in low- and middle-income countries. This calls for controlled longitudinal studies to characterize the dual burden of HIV and noncommunicable diseases. Furthermore, noncommunicable diseases need to be addressed holistically by both care and prevention, involving the psychosocial aspects that they impose on patients. This can only be delineated through further research on the complex interaction between HIV and noncommunicable diseases.

In conclusion, concomitant diabetes mellitus and HIV status disclosure to a close person were associated with poor blood pressure control among persons living with HIV who have hypertension. Therefore, subpopulations of persons living with HIV with comorbid hypertension and diabetes mellitus may require tailored approaches to achieve blood pressure targets.

## Summary

### What is known about this topic

- HIV has primarily become a chronic disease due to the advent of antiretroviral therapy
- Hypertension is the single most important modifiable risk factor for cardiovascular disease

### What this study adds

- Demonstrates the benefit of integrating blood pressure management in public HIV health programs
- Evaluates which factors are associated with blood pressure control in integrated HIV-hypertension care

## Data Availability

The datasets used and analyzed that support the findings of this study are available from the corresponding author upon reasonable request.
